# Efficacy of vestibular rehabilitation and its facilitating and hindering factors from real-world clinical data

**DOI:** 10.3389/fneur.2024.1329418

**Published:** 2024-02-29

**Authors:** Min-Ku Kim, So-Yeon Yun, Seonkyung Lee, Ja-Ok Lee, Soo-Yun Sung, Ju-Young Lee, Hyo-Jung Kim, Hye Youn Park, Jeong-Yoon Choi, Jae-Jin Song, Byung Yoon Choi, Ja-Won Koo, Ji-Soo Kim

**Affiliations:** ^1^Department of Neurology, Dizziness Center, Clinical Neuroscience Center, Seoul National University Bundang Hospital, Seongnam-si, Republic of Korea; ^2^Biomedical Research Institute, Seoul National University Bundang Hospital, Seongnam-si, Republic of Korea; ^3^Department of Psychiatry, Dizziness Center, Seoul National University Bundang Hospital, Seongnam-si, Republic of Korea; ^4^Department of Otorhinolaryngology-Head and Neck Surgery, Dizziness Center, Seoul National University Bundang Hospital, Seongnam-si, Republic of Korea

**Keywords:** vestibular rehabilitation, Vertigo, dizziness, vestibular disorders, psychological distress

## Abstract

**Background and purpose:**

Customized vestibular rehabilitation improved dizziness and imbalance in several randomized controlled trials. In the present study, we determined the efficacy of customized vestibular rehabilitation using real-world observational data.

**Methods:**

In this retrospective observational study, we recruited 64 patients (median age = 60, interquartile range = 48–66.3) who completed the customized vestibular rehabilitation from January to December 2022. The outcomes of rehabilitation were evaluated using the dizziness handicap inventory (DHI) or vestibular disorders activities of daily living scale (VADL). The factors associated with outcomes were assessed with a generalized linear model, of which covariates included patients’ age, sex, duration of illness, type of vestibular disorders, initial DHI and VADL scores, exercise compliance, and initial hospital anxiety and depression scale (HADS) scores.

**Results:**

After the median of 6 (4–6) weeks of rehabilitation, DHI and VADL scores significantly improved in patients with either peripheral or central vestibular disorders (Wilcoxon signed-rank test, *p* < 0.05). The initial DHI and VADL scores showed a positive while the sum of HADS scores showed a negative correlation with the outcome. In contrast, the age, sex, duration of illness, types of vestibular disorders, and exercise compliance did not affect the outcome.

**Discussion and conclusion:**

Customized vestibular rehabilitation is effective for central as well as peripheral disorders, especially when the symptoms are severe and the psychological distress is mild.

## Introduction

Customized vestibular rehabilitation, an individualized exercise-based treatment program, is designed for patients having physical or psychological disabilities due to vestibular disorders (1). It improves dizziness and imbalance by facilitating vestibular compensation mechanisms, such as adaptation, substitution, and habituation (2). Currently, early initiation of customized vestibular rehabilitation is strongly recommended for the patients with unilateral or bilateral peripheral vestibular disorders (3). Several systematic reviews supported the efficacy of vestibular rehabilitation in uni- or bilateral vestibulopathy (4–6), post-acoustic neurectomy (7), and cerebral or labyrinthine concussion (8, 9). It has also been adopted for patients with persistent postural-perceptual dizziness or other functional dizziness (1, 10, 11). The efficacy is also being reported in vestibular migraine and other central vestibular disorders (12–15). What is known more is that vestibular rehabilitation is effective regardless of patients’ age, symptom duration and intensity, but is more effective when patients do not have psychological distress such as anxiety and depression (2, 16–21).

Research-based clinical trials have shown clear benefits of customized vestibular rehabilitation, but its efficacy requires further support using the data acquired from routine clinical practice. Unlike clinical trials, there can be heterogeneities of the patients participating in vestibular rehabilitation in routine clinical practice, in terms of cause, duration, and severity of vestibular disorders as well as their comorbidities. These factors may promote or hinder the efficacy of vestibular rehabilitation. Based on these backgrounds, the present study evaluated the efficacy of customized vestibular rehabilitation using real-world observational data.

## Methods

### Study design, population, and ethical approval

This retrospective observational study involved patients undergoing customized vestibular rehabilitation at our dizziness center. The customized vestibular rehabilitation program in our center, presented in detail in the following subsection, was started in March 2019. For the 2 years up to December 2021, we improved the program regarding patient enrollment, contents and materials, monitoring patient participation, and outcome capture. Thereafter, we systematically registered patients’ data participating in the program since January 2022. In the present study, we recruited patients’ data from January 2022 to December 2022. Initially, 75 patients were screened, but 64 patients (35 males) were finally included (median age = 60, interquartile range = 48–66.3), excluding nine drop-outs and two missing outcomes. The included patients underwent vestibular function tests initially, using three-dimensional video-oculography, video head-impulse test, bithermal caloric tests, rotation chair test, cervical and ocular vestibular evoked myogenic potentials, and pure tone audiometry, selectively according to the clinical necessities. This study followed the tenets of the Declaration of Helsinki, and the Institutional Review Board of Seoul National University Bundang Hospital approved the study protocol and waived acquiring written consents from each patient (B-2303-814-101).

### Customized vestibular rehabilitation program

The vestibular rehabilitation exercise aimed to facilitate vestibular recovery and central compensation (2). Recently, the Korean Balance Society proposed the general guideline for customized vestibular rehabilitation, which applies adaptation, habituation, and substitution exercises at the perceptual, ocular motor, and postural levels. According to the guideline, we designed the vestibular rehabilitation program suitable to our center, consisting of ocular, postural, and habituation exercises (22). The ocular exercise consisted of 0.5 and 1 Hz horizontal and vertical vestibulo-ocular reflex (VOR), VOR following saccades, and active eye-head saccades exercises. The two formers were set to facilitate adaptation, and the latter was set to promote substitution. In the VOR exercise, patients were instructed to maintain their line of sight on a specific target while shaking their heads at specific frequencies guided by metronome auditory cue. In the VOR following saccades, two horizontal or vertical targets were presented. Patients alternated between these targets using auditory cues, initiating saccades first, followed by head movements at 1-s intervals. Finally, the active eye-head saccades closely resembled the VOR following saccades, but we allowed patients to execute eye and head movements freely and swiftly, guided by their intentions. The course varied among patients, but it generally began with 0.5 Hz VOR and VOR following saccade exercises in the first session, progressing to 1 Hz VOR and active eye-head saccade exercises in the subsequent sessions. The postural exercise was stratified into standing on the floor with back support, standing on a foamed matrix, and walking, and was designed to be performed together with 0.5 Hz horizontal and vertical VOR exercises. The habituation exercise was the repetitive exposure of the positions and stimuli identified to provoke dizziness and vertigo in the motion sensitivity quotient (MSQ) (23).

In our center, the customized vestibular rehabilitation was a referral-based outpatient program comprising four sessions at two-week intervals supervised by neuro-otologists and specialized clinical nurses. During the sessions, we evaluated patients’ functional status and exercise compliance, prescribed and updated customized exercises, and instructed and trained patients to do the exercise at home correctly. Patients were instructed to exercise thrice daily, for 40 min each time. To enhance participation, we provided an exercise diary, educational videos for exercise at every session, and alarm calls 1 week before the next session. Safety is a significant concern, so instructors emphasized to the patients to avoid and prevent fall-related injuries during the exercise.

Patients have already had the laboratory vestibular function tests required by the referring clinicians. Therefore, in the rehabilitation session, we only assessed the functional status of patients at the first and last sessions through dizziness handicap inventory (DHI) (24), vestibular disorders activities of daily living scale (VADL) (25), hospital anxiety and depression scale (HADS) (26), MSQ (23), Romberg test, and 50-step test (27). DHI which allows evaluating self-perceived handicaps due to dizziness contains 25 items with scoring 0 (lowest), 2 (medium), or 4 (highest) for each item and becomes 100 points in maximum (i.e., 4 × 25). A higher total score indicates a severer handicap due to dizziness (24). VADL was developed to evaluate patients with dizziness and vertigo by modifying the activities of daily living scale, specifically focusing on essential functional skills, mobility, and instrumental skills, rather than the quality of life *per se* (25, 28). It consists of 28 items of which each being scaled from 1 to 10 points and the maximum is 280 points (i.e., 10 × 28) (25). HADS evaluates anxiety and depression in the setting of an outpatient clinic and has 7 items each for anxiety and depression assessments, intending to measure mutually exclusive levels of anxiety and depression (26). However, its ability to distinguish between the constructs of anxiety and depression is obscure (29), thereby we used the total score of HADS. The compliance was assessed using the patients’ exercise diaries. From that, we could calculate a compliance score as the ratio of exercise sessions performed to given exercise sessions during the session interval. For example, when the patient was prescribed 1 Hz-VOR, standing on a formed matrix, and habituation exercises three times a day for 2 weeks, the denominator was 126 [= 3 (types of exercise) × 3 (times per day) × 14 (days)]. If the patients filled 63 sessions, the exercise compliance was 50%.

### Study outcomes

In the current study, customized vestibular rehabilitation outcomes were the improvement of subjective dizziness and functional status in daily activities, assessed by the improvement of DHI or VADL scores. However, instead of simply using the difference between pre-and post-DHI and VADL scores as the outcomes, we developed and used the efficacy index to minimize the basal effect of DHI and VADL scores, calculated as follows.


$$\begin{array}{l} \mathrm{Efficacy}\;\mathrm{index}\;\left(\%\right)=\\ \frac{Pre\;DHI\;\mathrm{or}\;\mathrm{VADL}\;\mathrm{score}-\mathrm{Post}\;DHI\;\mathrm{or}\;\mathrm{VADL}\;\mathrm{score}}{Pre\;DHI\;\mathrm{or}\;\mathrm{VADL}\;\mathrm{score}}\times 100 \end{array}$$

### Statistical analysis

All data are presented as a median with interquartile range and as number and percentage for categorical variables. For the comparison of DHI and VADL scores before and after the program, the Wilcoxon signed-rank test was adopted, while an improvement of DHI and VADL scores between peripheral and central vestibular disorders was evaluated by the Mann–Whitney U-test. To disclose the factors associated with the efficacy of customized vestibular rehabilitation (efficacy index), we examined patients’ age, sex, initial DHI and VADL scores, duration of illness, types of vestibular disorders (central vs. peripheral), exercise compliance scores, and initial HADS scores using a generalized linear model. The normality of error in the model was tested by Kolmogorov–Smirnov test. The statistical level for significance was set to less than 0.05. All data processing and statistical analyses were performed using MATLAB software (The MathWorks, Natick, MA, United States).

## Results

### Baseline characteristics of included patients

Of 64 patients, 35 (55%) had peripheral lesions. Twenty-nine had acute or chronic unilateral peripheral vestibulopathy (previously termed “vestibular neuritis”), 2 had bilateral vestibulopathy, 3 had peripheral vestibulopathy from Meniere’s disease (1 received vestibular neurectomy and 2 showed chronic dizziness without recent vertigo attack), and one had other peripheral lesions. The central lesions (*n* = 29) included infratentorial strokes (*n* = 8), persistent postural-perceptual dizziness (*n* = 7), cerebellar tumors (*n* = 6), cerebellar ataxia or multisystem atrophy (*n* = 6), and other central causes (*n* = 2).

The median interval from symptom onset to evaluation was 4 months (interquartile range = 2–10). The initial DHI, VADL, and sum of HADS scores were 50 (27.5–68.5), 78 (54–146.5), and 17 (9.8–22), respectively. The compliance score was 64.6 (40.8–83.9). The number of participations in the outpatient rehabilitation session was 4 (3–4), and the duration of exercise was 6 (4–6) weeks.

### Outcomes of vestibular rehabilitation and factor associated with the efficacy

**Figure 1:**
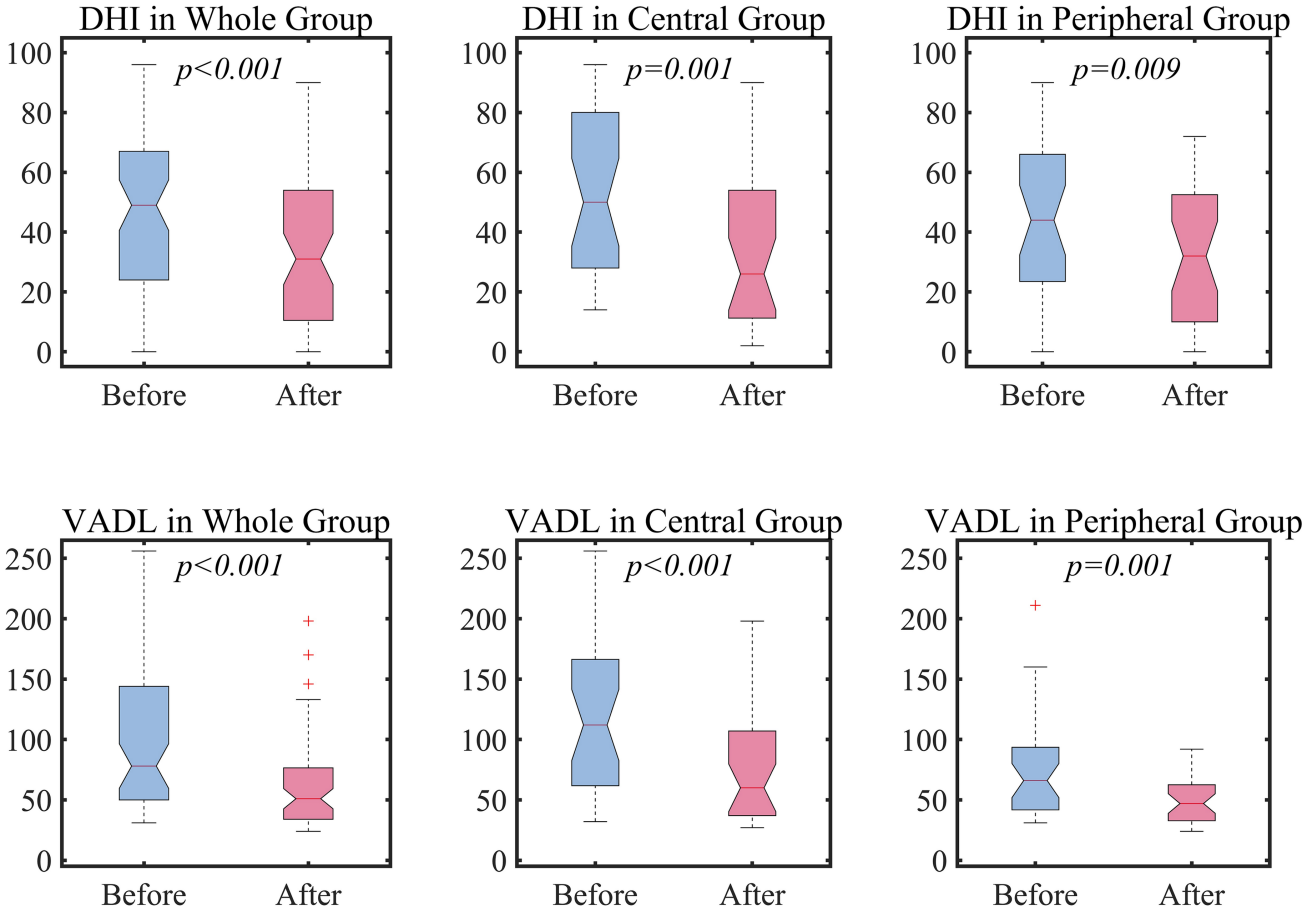
The efficacy of vestibular rehabilitation. For the statistical comparison of DHI and VADL scores between before and after vestibular rehabilitation, Wilcoxon signed-rank test was adopted. DHI, Dizziness handicap inventory; VADL, Vestibular activity of daily living.

After customized vestibular rehabilitation, the DHI and VADL scores were 36 (12–54) and 53 (34–78) with a significant statistical difference from the baseline scores (both *p* < 0.05, Wilcoxon signed-rank test, Figure 1). These changes were observed both in the peripheral and central groups. Of note, seven patients had no benefit or worsening after vestibular rehabilitation. These patients included four in the peripheral group (three with acute or chronic unilateral vestibulopathy and 1 with peripheral vestibulopathy from Meniere’s disease), and three in the central group (two with cerebellar ataxia and one with cerebellar tumor). All these patients had suffered from dizziness for more than 4 months. The changes in the DHI and VADL scores did not differ between the central and peripheral groups (*p* = 0.29 and 0.13, Mann–Whitney U-test).

**Figure 2:**
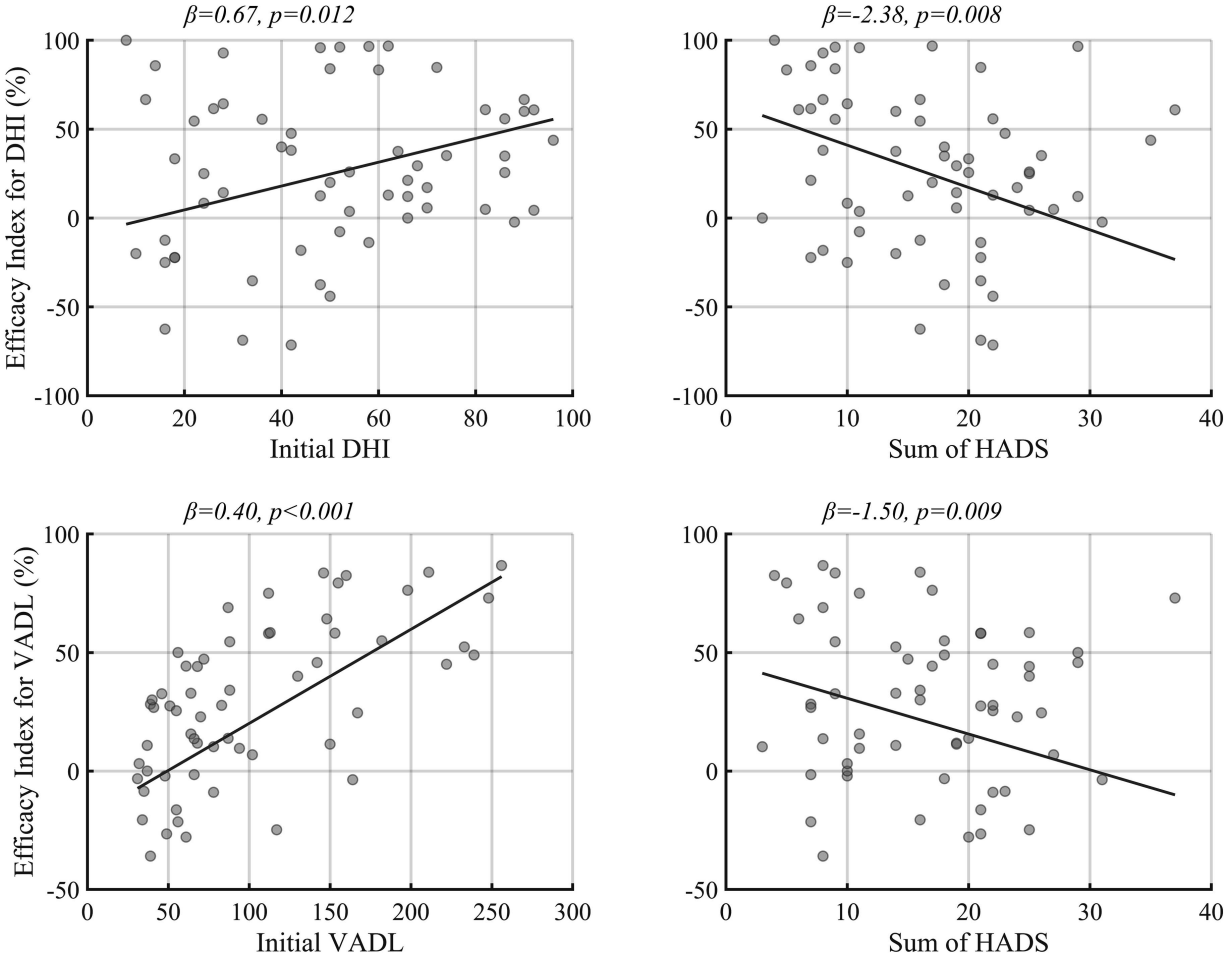
The factors associated with vestibular rehabilitation efficacy. The regression slope was drawn by setting the other covariates of regression model 1 and 2 to male, central vestibular disorder, and mean value of DHI, VADL, compliance, duration, and sum of HADS. HADS, Hospital anxiety and depression scores. Other abbreviations were identical to those of Figure 1.

Table 1 and Figure 2 summarize the results of generalized linear model analyses, which attempted to figure out the factors associated with the efficacy of vestibular rehabilitation (the efficacy index). The initial DHI and VADL scores showed a positive correlation with the efficacy indexes, implying that the patients with higher DHI and VADL scores had more benefits from customized vestibular rehabilitation. In contrast, the sum of HADS scores was negatively correlated with the DHI and VADL efficacy indexes. The age and sex of the patients, duration of illness, types of vestibular disorders, and exercise compliance scores did not correlate with the efficacy indexes.

**Table 1:** Factor associated with the efficacy of vestibular rehabilitation.

	Estimated β (95% CI)	*p*-value
Model 1 (outcome: efficacy index for DHI)		
Intercept	37.08 (−22.93 to 97.08)	0.231
Sex	7.65 (−15.42 to 30.72)	0.519
Age	0.03 (−0.83 to 0.89)	0.951
Duration	−0.02 (−0.19 to 0.15)	0.854
Compliance	−0.12 (−0.57 to 0.34)	0.618
Initial DHI*	0.67 (0.16 to 1.18)	0.012
Sum of HADS*	−2.38 (−4.08 to −0.69)	0.008
Group	−7.27 (−30.85 to 16.31)	0.548
Model 1 with interaction (outcome: efficacy index for DHI)		
Intercept	91.82 (−38.11 to 145.52)	<0.001
Initial DHI	−0.47 (−1.44 to 0.51)	0.354
Sum of HADS*	−6.33 (−9.60 to −3.05)	<0.001
Interaction*	0.068 (0.017 to 0.118)	0.011
Model 2 (outcome: efficacy index for VADL)		
Intercept	1.73 (−42.85 to 46.31)	0.940
Sex	5.17 (−10.66 to 20.99)	0.525
Age	−0.17 (−0.77 to 0.44)	0.589
Duration	0.04 (−0.08 to 0.16)	0.524
Compliance	0.21 (−0.1 to 0.52)	0.181
Initial VADL*	0.40 (0.25 to 0.54)	<0.001
Sum of HADS*	−1.51 (−2.59 to −0.42)	0.009
Group	4.18 (−12.72 to 21.09)	0.630
Model 2 with interaction (outcome: efficacy index for VADL)		
Intercept	−0.18 (−35.97 to 35.62)	0.992
Initial VADL*	0.48 (0.19 to 0.77)	0.002
Sum of HADS	−0.80 (−2.86 to 1.27)	0.453
Interaction	−0.01 (−0.02 to 0.01)	0.468

Generalized linear models with linear fit were adopted for the statistics. CI, confidence interval; DHI, Dizziness handicap inventory; VADL, Vestibular activity of daily living; HADS, Hospital anxiety and depression scores.Asterisks denote variables with statistical significance.

### Relationship of initial disability and anxiety-depression on the efficacy outcome

To detail the relationship of initial disability and anxiety-depression, further analyses were adopted using a subset of the generalized linear model. This approach included only the initial scores of DHI and VADL, the total HADS scores, and their interaction terms as factors. The analyses revealed a significant interaction between the initial DHI and the total HADS scores in terms of rehabilitation efficacy (interaction *p* = 0.011). However, there was no significant interaction between the initial VADL and the total HADS scores (Table 1).

## Discussion

This study evaluated the efficacy of customized vestibular rehabilitation implemented in a referral-based tertiary hospital. Customized vestibular rehabilitation in real-world clinical practice improved subjective handicap and functional skills regardless of the types of vestibular disorders, as shown in previous clinical trials (2). Furthermore, this study demonstrated that patients with severe subjective handicaps and functional impairments got more benefits from vestibular rehabilitation, while those with psychological distresses had lesser benefits.

Customized vestibular rehabilitation is designed to improve dizziness and related functional disabilities. So far, it has been tested in various vestibular disorders with several outcomes and proven its efficacy mostly in peripheral vestibular disorders. The efficacy for the subjective handicaps and functional impairments assessed with DHI also appears real in several studies. In the Cochrane data review, patients with unilateral peripheral vestibulopathy comprised of Meniere’s disease and acute or subacute vestibular neuritis got significant benefits on DHI scores from vestibular rehabilitation in the meta-analysis subjected for 535 patients from 5 studies (4). Although there were heterogeneities in etiology and duration of illness among studies, the mean DHI improvements in vestibular rehabilitation ranged from 5 to 42 points and differed significantly from the mean DHI changes without rehabilitation, which ranged from −2 to 21 points. In our study, the peripheral group showed 10 points of median DHI improvement, comparable to observations in the previous randomized control studies. Meanwhile, the study evaluating the efficacy using VADL improvement in peripheral vestibular disorders was sparse (30). The effect of vestibular rehabilitation for central vestibular disorders, such as stroke, traumatic brain injury, persistent postural-perceptual dizziness, and vestibular migraine, has also been tested (8, 9, 12–15, 31). The results were promising, but more robust evidence needs to be supplemented. In that view, the present study sheds light on the possible efficacy of customized vestibular rehabilitation on central vestibular disorders, given the comparable observed efficacy between central and peripheral disorders. The observed benefits in patients with central lesions might seem contradictory in light of the traditional mechanism of vestibular rehabilitation. However, in cases of central lesions, the compensatory circuit might not be directly involved, or if it is involved, it may still have the potential for recovery unless the lesion is of a widespread and progressive nature. Our study, therefore, suggested that customized vestibular rehabilitation could effectively manage patients with both peripheral and central vestibular disorders.

Then, the clinician would have questions in implementing vestibular habilitation: who can get more or less benefits from vestibular rehabilitation? The answers to these questions will help provide appropriate clinical guidance. In this study, we tried to answer the question by analyzing the association between the efficacy of rehabilitation and patients’ factors, including age, sex, symptom severity, duration of illness, types of vestibular disorders (central vs. peripheral), exercise compliance, and psychological distress such as anxiety and depression. In terms of symptom severity, a previous study insisted the effect of vestibular rehabilitation was irrelevant and rather tended to decrease with an increase in severity (21). However, the study only adopted zero-to-five-point disability scores to assess vestibular rehabilitation efficacy rather than sophisticated questionnaire such as DHI or VADL scores we adopted. In the present study, the symptom severity had a clear positive correlation with the efficacy of vestibular rehabilitation, implying patients suffered more from subjective handicaps and functional impairments are subjected to the beneficiary. We believe this finding would become more robust by adopting the efficacy indexes to minimize the basal effect of spontaneous recovery. Therefore, if capable, vestibular rehabilitation should be recommended, especially in patients having severe symptoms.

Regarding psychological comorbidity, we noted that patients with higher anxiety and depression would benefit less. This result was in line with the previous suggestions that vestibular rehabilitation is more effective in patients without psychological distress, such as anxiety and depression (19, 20). What should not be misinterpreted, however, is that vestibular rehabilitation is still beneficial for those with psychological distress. There has been just variance in recovery depending on the severity of the psychological distress (19). Therefore, appropriate psychological intervention may be warranted for those with severe psychological distress to increase the efficacy of vestibular rehabilitation. Of interest, the levels of anxiety and depression appeared to improve with vestibular rehabilitation (32, 33), suggesting vestibular rehabilitation can help reduce psychological distress associated with vestibular disorders. Taken together, the assessment and treatment of psychological distress at the very first of vestibular rehabilitation might bring better outcomes.

The subset analyses exploring the relationship between initial disability and anxiety-depression on the efficacy outcome revealed inconsistent findings. Interestingly, when considering the DHI scores, the direct effect of initial severity on the efficacy of rehabilitation became insignificant. Instead, the interaction between DHI and HADS scores indicated that for patients with higher levels of anxiety and depression, the initial level of disability had a more pronounced positive impact on rehabilitation efficacy. However, generalization of this suggestion may be limited, as this pattern was not observed when severity was assessed using the VADL scores.

We evaluated other demographic features possibly linked with the efficacy of vestibular rehabilitation, and the results provided additional information. First, age and sex have not associated with the outcome, as is previously suggested (16, 21). Second, in general, implementing vestibular rehabilitation in the acute stage can bring maximal functional improvement but also shows favorable outcomes in the chronic stage (2, 17, 18). The present study included heterogeneous populations in the duration of illness, from within days to more than tens of years, and revealed the duration of illness did not relate to the outcomes, suggesting its implementation at any given time. Third, there would be a question of whether vestibular rehabilitation is equally effective both in central and peripheral disorders. Previous observation suggested that vestibular rehabilitation outcomes are independent of the type of vestibular disorder (2), though central lesions needed more extended treatment to gain the effect (18, 34, 35), and pure central lesions were noted to get a better outcome than mixed central and peripheral lesions (20). However, there has yet to be a comparative study about this issue. The present study demonstrated that the efficacy of vestibular rehabilitation was unrelated to the type of vestibular lesions. Even though the effect would vary according to the pathology of lesions and be minimal in patients with degenerative and progressing lesions, this finding encourages the adoption of vestibular rehabilitation in patients with central vestibular disorders. Lastly, we did not find any association between exercise compliance and the efficacy of vestibular rehabilitation. Faithful participation leads to a better outcome in common sense, but there was no significant association between the two. One possible explanation is the homogeneity of included patients in compliance since we only analyzed the patients who completed the program. Other than that, the improvement after the rehabilitation may affect compliance. For instance, patients having a more remarkable improvement in the early stage may participate unfaithfully, thereby contributing to obscure the association. Further studies are warranted to resolve this issue.

This study has several limitations. First, the study design, in which patients who completed the program and had outcomes were recruited at a single center and analyzed retrospectively, inevitably exposed to selection bias. Second, the effect of vestibular rehabilitation itself could not be ascribed due to lacking a control group. Indeed, the effect of natural recovery would have influenced the rehabilitation outcomes. However, the median interval from symptom onset to initial rehabilitation was 4 months, indicating that most patients were in the chronic stage of their illness. Considering that symptoms mostly recover during the acute to subacute stages, the absence of a control group would not greatly hinder the interpretation of the study results. Additionally, since the effectiveness of vestibular rehabilitation has been well established, there were limitations in designing a control group within the context of real-world practice for this study. Therefore, the goal of the present study was to examine the efficacy of vestibular rehabilitation in real-world clinical practice and to identify factors associated with the outcomes. Third, the outcomes included subjective handicaps and functional impairments, while the physical findings and laboratory assessment were not included. Lastly, the number of included patients was small, and the causes of vestibular disorders were diverse, particularly within the central group, thereby limiting the derivation of meaningful results. Therefore, to consolidate the present study’s findings, a large number of patients with multi-dimensional outcome assessments are warranted in future studies.

## Conclusion

In conclusion, customized vestibular rehabilitation should be recommended for patients with vestibular disorders, at any stage and for both peripheral and central lesions. The benefits would likely be more significant for severe symptoms but lesser for severe psychological distress.

## Data availability statement

The raw data supporting the conclusions of this article will be made available by the authors, without undue reservation.

## Ethics statement

The studies involving humans were approved by the Institutional Review Board of Seoul National University Bundang Hospital. The studies were conducted in accordance with the local legislation and institutional requirements. Written informed consent from the patients or patients legal guardian/next of kin was not required to participate in this study in accordance with the national legislation and the institutional requirements.

## Author contributions

M-KK: Data curation, Formal analysis, Writing – original draft. S-YY: Conceptualization, Data curation, Formal analysis, Investigation, Methodology, Software, Writing – original draft. SL: Data curation, Investigation, Methodology, Validation, Writing – review & editing. J-OL: Data curation, Investigation, Methodology, Writing – review & editing. S-YS: Writing – review & editing. J-YL: Supervision, Writing – review & editing, Data curation, Formal analysis, Methodology. H-JK: Conceptualization, Data curation, Methodology, Writing – review & editing. HP: Conceptualization, Data curation, Formal analysis, Investigation, Methodology, Software, Writing – review & editing. J-YC: Conceptualization, Data curation, Formal analysis, Project administration, Supervision, Visualization, Writing – original draft, Writing – review & editing. J-JS: Conceptualization, Data curation, Formal analysis, Investigation, Methodology, Supervision, Writing – review & editing. BC: Data curation, Investigation, Methodology, Software, Supervision, Writing – review & editing. J-WK: Data curation, Investigation, Methodology, Software, Supervision, Writing – review & editing. J-SK: Data curation, Investigation, Methodology, Software, Supervision, Writing – review & editing.
